# Effect of polydeoxyribonucleotide as a potential therapeutic agent to enhance the healing of Crohn’s perianal fistula

**DOI:** 10.1093/ibd/izag047

**Published:** 2026-04-11

**Authors:** Hyeon Kyeong Kim, Yong Sik Yoon, Jong Lyul Lee, Eun Jung Park, Min Hyun Kim, Young Il Kim, Chan Wook Kim, In Ja Park, Seok-Byung Lim, Chang Sik Yu

**Affiliations:** Department of Surgery, College of Medicine, St. Vincent’s Hospital, The Catholic University of Korea , Address 93, Jungbu-daero, Paldal-gu, Suwon-si, Gyeonggi-do, South Korea; Division of Colon and Rectal Surgery, Department of Surgery, University of Ulsan College of Medicine, Asan Medical Center , Address 88 Olympic-ro 43-gil, Songpa-gu, Seoul 05505, South Korea; Division of Colon and Rectal Surgery, Department of Surgery, University of Ulsan College of Medicine, Asan Medical Center , Address 88 Olympic-ro 43-gil, Songpa-gu, Seoul 05505, South Korea; Division of Colon and Rectal Surgery, Department of Surgery, University of Ulsan College of Medicine, Asan Medical Center , Address 88 Olympic-ro 43-gil, Songpa-gu, Seoul 05505, South Korea; Division of Colon and Rectal Surgery, Department of Surgery, University of Ulsan College of Medicine, Asan Medical Center , Address 88 Olympic-ro 43-gil, Songpa-gu, Seoul 05505, South Korea; Division of Colon and Rectal Surgery, Department of Surgery, University of Ulsan College of Medicine, Asan Medical Center , Address 88 Olympic-ro 43-gil, Songpa-gu, Seoul 05505, South Korea; Division of Colon and Rectal Surgery, Department of Surgery, University of Ulsan College of Medicine, Asan Medical Center , Address 88 Olympic-ro 43-gil, Songpa-gu, Seoul 05505, South Korea; Division of Colon and Rectal Surgery, Department of Surgery, University of Ulsan College of Medicine, Asan Medical Center , Address 88 Olympic-ro 43-gil, Songpa-gu, Seoul 05505, South Korea; Division of Colon and Rectal Surgery, Department of Surgery, University of Ulsan College of Medicine, Asan Medical Center , Address 88 Olympic-ro 43-gil, Songpa-gu, Seoul 05505, South Korea; Division of Colon and Rectal Surgery, Department of Surgery, University of Ulsan College of Medicine, Asan Medical Center , Address 88 Olympic-ro 43-gil, Songpa-gu, Seoul 05505, South Korea

**Keywords:** Crohn’s disease, perianal fistula, polydeoxyribonucleotide, fistula/surgery, wound healing

## Abstract

**Background:**

The treatment of Crohn’s perianal fistula remains challenging owing to its recurrent nature and resistance to complete healing. This study aimed to assess the postoperative outcomes, including fistula closure rates, following Crohn’s perianal fistula surgery, with and without polydeoxyribonucleotide administration.

**Methods:**

A retrospective review was conducted on patients who underwent fistula closure operations for Crohn’s perianal fistula at a single tertiary center in Seoul, Korea, between January 2018 and June 2024. Patients were divided into two groups based on the use of local polydeoxyribonucleotide (PDRN) injections during fistula closure operations, and clinical variables and postoperative outcomes were compared between the groups.

**Results:**

Among the 47 patients, 21 (44.7%) were assigned to the PDRN group and 26 (55.3%) were assigned to non-PDRN group. Baseline characteristics between the two groups showed no significant difference. During the mean follow-up of 30.4 months, patients in the PDRN group demonstrated a significantly higher complete fistula closure rate (70.0% vs 26.9% at 6 months, and 83.3% vs 46.2% at 12 months; *P* = .005), and a shorter time from surgery to complete closure (3.3 ± 2.6 months vs 5.9 ± 3.8 months; *P* = .044), compared with the non-PDRN group. Multivariate analysis revealed PDRN as an independent factor significantly associated with Crohn’s perianal fistula closure (HR 2.336; 95% CI, 1.092-4.997, *P* = .029).

**Conclusions:**

PDRN is a potential therapeutic agent that presents a viable option for Crohn’s perianal fistula surgery, improving complete fistula closure rate through a safe, cost-effective, and relatively simple procedure.

## Graphical abstract

**Graphical Abstract:**
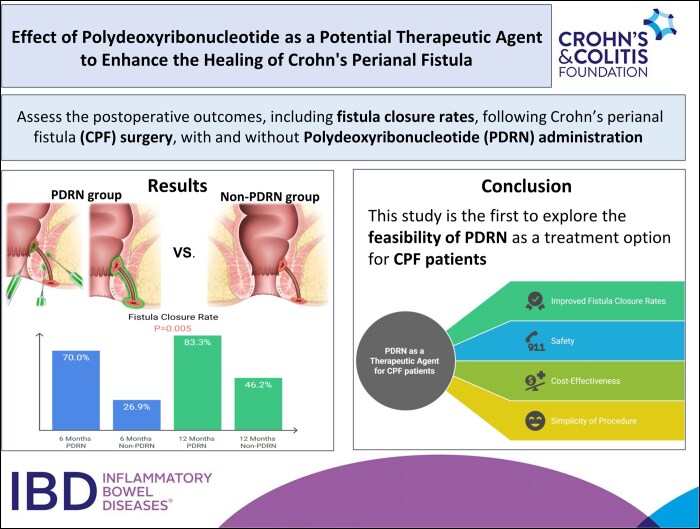

Key Messages *What is already known?*

The treatment of Crohn’s perianal fistula (CPF) remains a significant clinical challenge, necessitating surgical interventions combined with effective local injection strategies to optimize the treatment of complex CPF.

*What is new here?*

Our study is the first to demonstrate the feasibility of polydeoxyribonucleotides (PDRNs) as a potential agent to enhance fistula closure rates following CPF surgery, emphasizing their safety, practicality, and cost-effectiveness.

*How can this study help patient care?*

Given the high recurrence rate and refractory nature of CPF, PDRNs may serve as a viable local injection therapy for CPF patients, offering a cost-effective and convenient alternative to adipose-derived stem cells, which have demonstrated the highest success rates as local therapeutic agents in CPF surgery.

## Introduction

Crohn’s disease (CD) is a lifelong relapsing systemic condition resulting from an autoimmune disorder that affects the gastrointestinal tract, extending from the mouth to the anus. The associated transmural inflammation compromises the integrity of the intestinal mucosa, leading to the formation of abscesses and fistulas.^1^ A perianal fistula is one of the most common complications of CD, with an estimated lifetime risk of occurrence ranging from 13% to 28%.^2^ The presence of a Crohn’s perianal fistula (CPF) significantly reduces patients’ quality of life by causing pain, perianal discharge, and impairment of physical and sexual functions.^3^ Recent consensus statements have emphasized that CPF is a heterogeneous condition requiring individualized management based not only on fistula anatomy, but also on disease activity.^3^^,^^4^ Contemporary management of CPF has evolved toward a multidisciplinary, treat-to-target strategy integrating optimized medical therapy with timely surgical intervention. Within this framework, anti-tumor necrosis factor (anti-TNF) agents remain the cornerstone of first-line medical therapy, improving complete remission rates.^5^^,^^6^ However, achieving complete fistula closure in CPF remains a considerable challenge, and recurrence rates remain high. This highlights the ongoing need for strategies aimed at improving surgical outcomes in patients with CPF.

In the context of the contemporary TOpClass-based management paradigm, patients classified as class 2a—characterized by chronic symptomatic fistulae suitable for repair after optimization of medical therapy—may represent a clinically relevant subgroup in whom adjunctive regenerative strategies could be considered.^3^^,^^4^ In this setting, surgical management typically involves examination under anesthesia, adequate drainage of sepsis, and selective reparative procedures. To further enhance surgical outcomes, various local adjunctive therapies have been explored, including fibrin glue, intralesional anti-TNF injections, and mesenchymal stem cell therapy.^5^^,^^7^ Various agents, including anti-TNF agents, fibrin glue, and mesenchymal stem cells, have been explored,^8–11^ with stem cell therapy demonstrating the highest success rates.^11^ Allogeneic adipose-derived stem cells (ASCs) are commonly used in Europe, whereas autologous ASCs are preferred in other countries, such as Korea and the United States.^12^ However, stem cell therapy remains expensive and involves complex procedures for harvesting autologous ASCs. Therefore, developing more cost-effective and readily available surgical treatment options for complex CPF is necessary.

Polydeoxyribonucleotide (PDRN), extracted from trout sperm, is a novel agent that promotes wound healing and tissue repair by enhancing angiogenesis and exerting anti-inflammatory effects.^13^ Composed of deoxyribonucleotide polymers, PDRN is degraded by active enzymes in the cell membrane, yielding purine and pyrimidine deoxynucleosides and deoxyribonucleotides that promote cell proliferation.^14^^,^^15^ Additionally, PDRN functions as an anti-inflammatory agent by suppressing the release of inflammatory cytokines from macrophages.^13^ Because of these effects, PDRN has been widely adopted in various fields requiring wound healing and tissue regeneration, including burn injury, hair loss, diabetic foot ulcers, pressure ulcers, and autologous skin grafts.^14^^,^^16–20^ Given the high recurrence rate and intractable nature of CPF, identifying cost-effective and convenient alternatives with an efficacy comparable to that of ASCs is necessary. This study aimed to evaluate the potential benefits of PDRN in CPF by comparing postoperative outcomes, including fistula closure rates, between patients who were locally administered PDRN and those who were treated conventionally for the first time.

## Methods

### Patients and clinical characteristics

We retrospectively reviewed patients with CD who underwent fistula closure operations for CPF at Asan Medical Center in Seoul, Korea, between January 2018 and June 2024, with a minimum follow-up of 6 months. Since the selective use of PDRN began in 2021, patients were categorized into 2 groups based on the administration of local PDRN injections into tissues surrounding the fistula tract during surgery. Fistula closure operations were defined as all surgical procedures performed to achieve fistula-tract closure in a single step, including fistulotomy, fistulectomy, and internal opening closure (IOC). Patients undergoing seton placement were excluded to reduce heterogeneity; however, prior seton drainage was permitted, and seton removal was performed during IOC.

**Figure 1:**
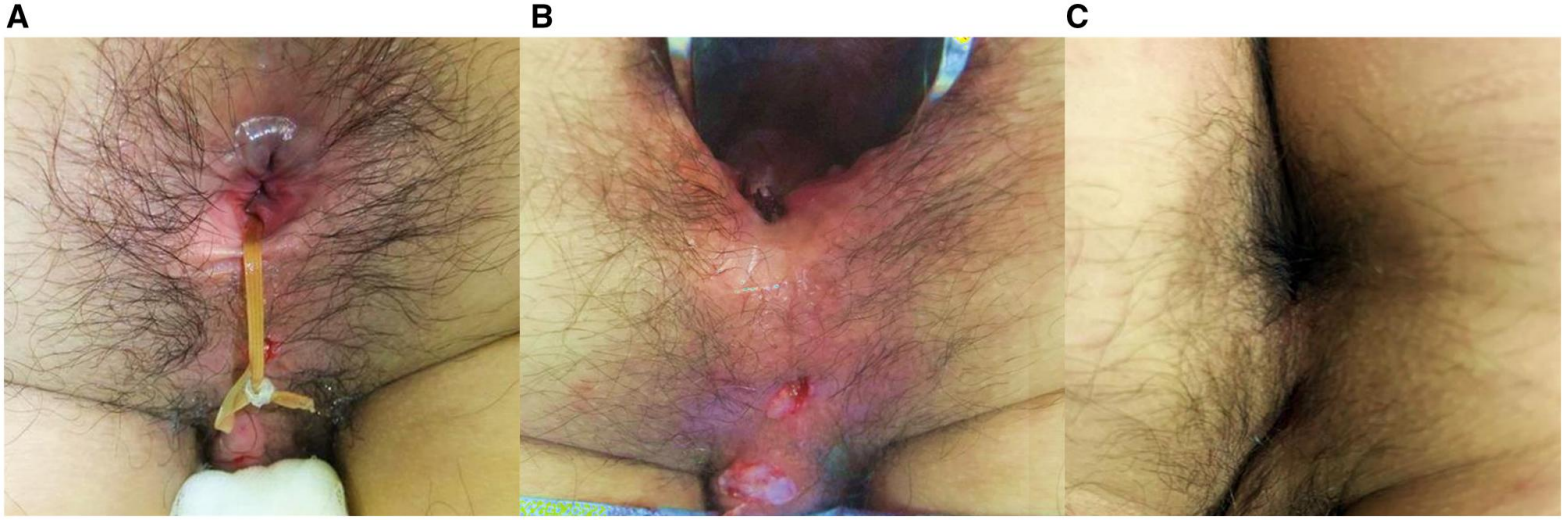
Clinical images from a case of polydeoxyribonucleotide (PDRN) administration, demonstrating postoperative healing. (A) Seton placement status before internal opening closure (IOC). (B) Immediate postprocedural status after IOC with PDRN injection. (C) Complete healing status after 4 months.

Postoperative outcomes included the rate of complete fistula closure, follow-up intervals, and time to complete fistula closure (closure time). Complete fistula closure was defined as total healing of the perianal wound or the absence of fluid discharge from the fistula following the fistula closure operation (Figure 1). For patients with multiple fistulas, fistula-related postoperative outcomes were assessed at the patient level (rather than per fistula), and complete closure was defined as resolution of all existing fistula tracts. Fistula recurrence was defined as the re-emergence of pain accompanied by perianal swelling or discharge during follow-up in patients who had previously achieved complete fistula healing.

Patient demographics, including age, sex, and smoking status, were analyzed. Fistula characteristics included fistula type (simple vs complex, single vs multiple) and the presence of proctitis or anorectal stricture. Anorectal stricture was defined as narrowing at the anal canal or distal rectum confirmed on examination under anesthesia that prevented or markedly limited passage of the examiner’s index finger and required dilation. Fistulas were classified according to Park’s criteria,^21^ with complex fistulas classified as transsphincteric, suprasphincteric, or extrasphincteric. Perioperative characteristics included the subclass of the Montreal classification, previous bowel resections, the number of previous fistula surgeries, disease duration, albumin levels, C-reactive protein (CRP) levels, Crohn’s Disease Activity Index, operation type, and perioperative CD medications. CRP was defined as the baseline value obtained preoperatively. Perioperative medications included antibiotics (eg, ciprofloxacin, metronidazole, cefpodoxime, vancomycin, and ampicillin/sulbactam), immunomodulators (eg, azathioprine, 6-mercaptopurine, and methotrexate), and biologics (eg, infliximab [Remicade; Janssen Biotech], adalimumab [Humira; AbbVie], ustekinumab [Stelera; Janssen Biotech], and vedolizumab [Entyvio; Takeda Pharmaceuticals]). Treatment with biologics was defined as at least 1 infusion occurring within 3 months before or after surgery, whereas treatment with immunomodulators was defined as administration within 2 months before or after surgery. Perioperative medical therapy was categorized as immunomodulator monotherapy or combination therapy with biologics and was assessed separately for preoperative and postoperative periods. The study protocol was approved by the Institutional Review Board of the Asan Medical Center (No. S2024-1574-0001). The requirement for informed consent was waived due to the retrospective nature of the study design.

### Surgical procedures

The type of surgery was determined by an experienced faculty surgeon (Y.S.Y.) with over 15 years of expertise, who performed approximately 50 CPF surgeries annually. Surgical decisions were based on factors such as the severity of inflammation and fistula complexity.

**Figure 2:**
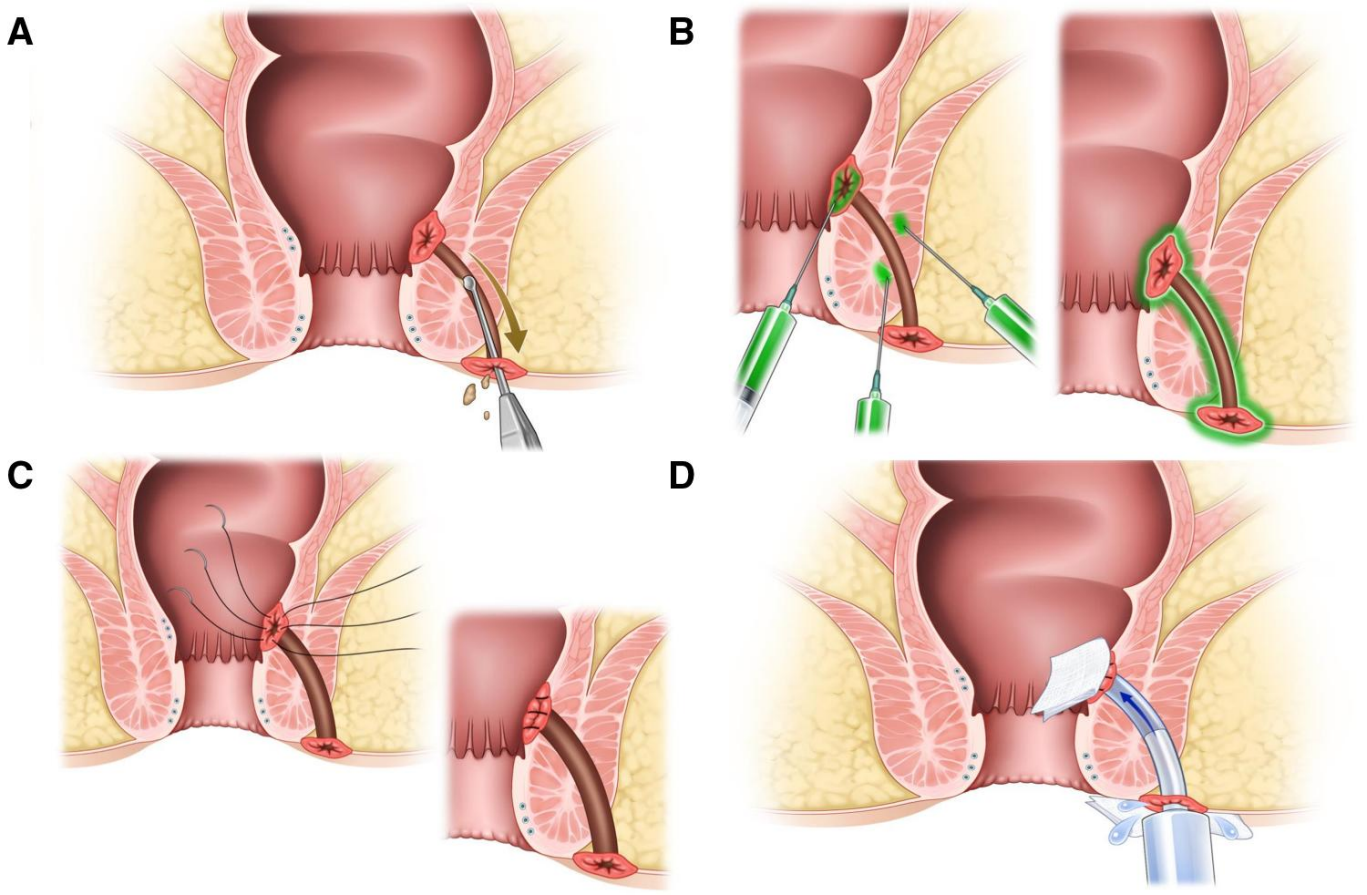
Procedure of polydeoxyribonucleotide (PDRN) injection during internal opening closure (IOC). (A) Curetting of the fistula tract. (B) PDRN injection around the fistula tract and internal opening. (C) IOC with interrupted sutures. (D) Water leak test.

All surgical procedures were performed under general anesthesia, with the patient in the prone jackknife position. In the absence of active proctocolitis, patients with simple transsphincteric, intersphincteric, or superficial fistulas underwent fistulotomy or fistulectomy.^22^ During these procedures, fistula tracts were probed through external openings. Superficial fistula tracts were either divided or excised under probe guidance, followed by curettage of the tracts and the surrounding infected tissues. In cases in which the fistula tract exhibited minimal inflammation or in which proctitis or inflammation had improved following bridging procedures such as seton placement, IOC was performed. This procedure involved thorough curettage to excise infected tissues surrounding the residual fistula tract, followed by closure of the internal opening using interrupted absorbable sutures. Subsequently, a water leakage test was conducted to confirm the integrity of the closure. Sterile water was injected into the fistula tract under pressure through the external opening using a syringe. The application of gauze to the external opening facilitated the maintenance of pressure during injection. The internal opening was covered with dry gauze, and the absence of moisture on the gauze was observed to determine the success of the closure (Figure 2). This approach aligns with methods employed in our previous studies on autologous ASC transplantation.^23^^,^^24^

In patients treated with locally injected PDRN, an additional injection of PDRN was administered around the internal opening and fistula tract. The dose was determined based on the length and diameter of the fistula, which were measured using a probe prior to injection. PDRN (Placentex Integro, 5.625 mg/3 mL [Mastelli Farmaceutica] or Vitaran, 5.625 mg/3 mL [BRPHARM]) was administered at a concentration of 0.6 ampoule (2 mL) per centimeter of the fistula tract. The dose was adjusted and increased in patients with advanced inflammation or higher complexity. In most cases, this standardized dosing was followed; however, in a small number of cases with pronounced local inflammation, the dose was increased at the surgeon’s discretion by approximately 10% to 20%, typically by adding about 1 additional ampoule. Based on the fistula tract measurement, 2 to 6 ampoules of PDRN were used for submucosal or intramuscular injection around the fistula tract and internal opening. The indication for definitive procedures such as fistulotomy, fistulectomy, and IOC was determined using the same standardized clinical criteria in both groups. For procedures involving IOC, PDRN was uniformly injected around the fistula tract immediately after curettage of the tract or adjacent infected tissues and just before suturing the internal opening (Figure 2). Local injection of PDRN before suturing the internal opening enables a uniform, multidirectional distribution around the fistula tract.

### Postoperative management and follow-up

After confirming the absence of medical complications during inpatient management, patients were discharged the next day and scheduled for an initial follow-up within 1 to 2 weeks. Subsequent follow-ups occurred every 1 to 2 months, with laboratory tests and physical examinations (including digital rectal examination when appropriate). Laboratory tests—complete blood count, basic chemistry panel (electrolytes and routine chemistry), and CRP—were routinely obtained at the during outpatient follow-up. If complete closure was achieved, follow-up at the colorectal surgeon’s outpatient clinic was considered unnecessary, and patients were advised to maintain regular follow-up care with their gastroenterologists.

### Statistical analyses

Continuous variables are presented as medians with ranges or as means with standard deviations. Comparisons of patient and clinical characteristics between the PDRN and non-PDRN groups were performed using Student’s *t* tests. Categorical variables were expressed as frequencies and percentages, and comparisons between the two groups were conducted using Pearson’s chi-square test or Fisher’s exact test. To evaluate the risk associated with complete fistula closure, hazard ratios (HRs) from univariate and multivariate analyses were calculated and assessed using Cox’s proportional hazards model. The cumulative rate of complete fistula closure was determined using the Kaplan-Meier method and compared between the two groups using the log-rank test. A subgroup analysis was performed to compare complete fistula closure between the two groups in patients who underwent either fistulotomy or fistulectomy, as well as in those who underwent IOC. Statistical significance was set at *P* < .05. All data analyses were performed using IBM SPSS Statistics for Windows, version 23 (IBM).

## Results

### Baseline characteristics

Among the 47 cases of fistula closure surgery performed for CPF, 21 (44.7%) were placed in the PDRN group and 26 (55.3%) were placed in the non-PDRN group. No significant differences were observed in patient demographics, fistula characteristics, or perioperative characteristics. Only a small proportion of patients had multiple fistulas (2/21 in the PDRN group and 2/26 in the non-PDRN group). In the PDRN group, the 2 patients had 2 and 3 tracts, respectively; in the non-PDRN group, both had 2 tracts. Accordingly, the complete closure rate among patients with multiple fistulas was 50% in each group (n = 1 of 2). The distribution of surgical procedures between the PDRN and non-PDRN groups was as follows: IOC was the most common surgery (n = 34), followed by fistulotomy (n = 10) and fistulectomy (n = 3) (Table 1). No significant differences were observed in the types of surgical procedures used between the two groups.

**Table 1:** Baseline patient characteristics.

	PDRN (n = 21)	Non-PDRN (n = 26)	*P* value
**Age, y**	28.3 (22.0-35.5)	26.9 (20.0-31.2)	.611
**Sex**			.734
** Female**	5 (23.8)	5 (19.2)	
** Male**	16 (76.2)	21 (80.8)	
**Smoking**	4 (19.0)	4 (14.8)	.715
**Fistula type**			.760
** Simple**	9 (42.9)	10 (38.5)	
** Complex**	12 (57.1)	16 (61.5)	
**Multiple fistulas**	2 (9.5)	2 (7.7)	1.000
**Proctitis**	1 (4.8)	0 (0.0)	.447
**Anorectal stricture**	2 (9.5)	3 (11.5)	.824
**Montreal classification**			
** Age at onset**			.496
** A1**	4 (19.0)	7 (26.9)	
** A2**	17 (81.0)	17 (65.4)	
** A3**	0 (0.0)	2 (7.7)	
** Location**			.275
** L1**	11 (52.4)	9 (34.6)	
** L2**	5 (23.8)	5 (19.2)	
** L3**	5 (23.8)	12 (46.2)	
** Behavior**			.112
** B1**	20 (95.2)	19 (73.1)	
** B2**	0 (0.0)	1 (3.8)	
** B3**	1 (4.8)	6 (23.1)	
**Previous bowel resection**	1 (4.8)	4 (15.4)	.362
**Prior seton placement**	10 (47.6)	10 (38.5)	.566
**Number of previous fistula operation times**	1.76 ± 1.17	1.27 ± 1.37	.379
**Disease duration, y**	4.24 ± 4.13	6.36 ± 7.21	.220
**Albumin, g/dL**	4.03 ± 0.35	3.93 ± 0.36	.337
**CRP, mg/dL**	0.77 ± 1.89	0.73 ± 1.00	.915
**CDAI**	105.04 ± 87.80	112.94 ± 118.98	.808
**Operation type**			.684
** Anal fistulotomy**	5 (23.8)	5 (19.2)	
** Anal fistulectomy**	2 (9.5)	1 (3.8)	
** IOC**	14 (66.6)	20 (76.9)	
**Medical treatment**			
**Preoperative**			
** Antibiotics**	6 (28.6)	4 (15.4)	.306
** IM**	15 (71.4)	20 (80.0)	.497
** Biologics**	8 (38.1)	11 (42.3)	.770
** IM monotherapy**	8 (38.1)	11 (42.3)	.770
** IM + biologic combination**	7 (33.3)	9 (34.6)	.927
**Medical treatment**			
**Postoperative**			
** Antibiotics**	9 (42.9)	5 (20.0)	.093
** IM**	18 (85.7)	23 (88.5)	.779
** Biologics**	11 (52.4)	15 (57.7)	.716
** IM monotherapy**	10 (47.6)	10 (38.5)	.566
** IM + biologic combination**	8 (38.1)	13 (50.0)	.557

Values are median (Q1-Q3) or n (%).

Abbreviations: CDAI, Crohn’s disease activity index; IM, immunomodulator; IOC, internal opening closure; CRP, C-reactive protein; PDRN, polydeoxyribonucleotide.

### Comparison of the rate of complete fistula closure

**Figure 3:**
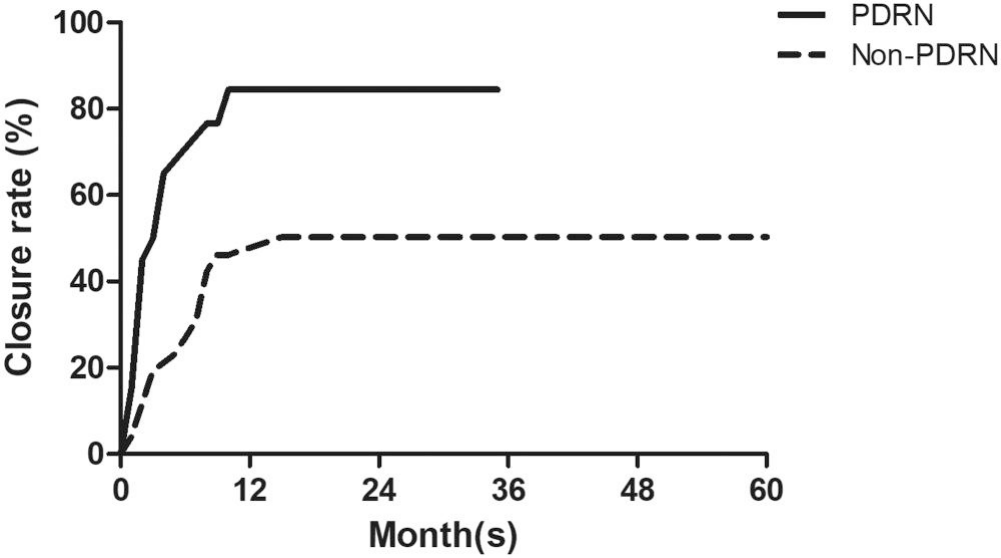
Kaplan-Meier curve comparing the complete fistula closure rates between the polydeoxyribonucleotide (PDRN) group and non-PDRN group.

During a mean follow-up of 30.4 months (19.2 months in the PDRN group vs 39 months in the non-PDRN group; *P* = .001), the PDRN group demonstrated significantly higher fistula closure rates compared with the non-PDRN group (70% vs 27% at 6 months and 83% vs 46% at 1 year; *P* = .005) (Table 2, Figure 3). Because all complete closures in the PDRN group occurred within 1 year, we focused on fixed time point estimates (6 months and 1 year) and time-to-event analyses to reduce bias from unequal follow-up. The duration from surgery to complete fistula closure was significantly shorter in the PDRN group than in the non-PDRN group (3.3 ± 2.6 months vs 5.9 ± 3.8 months; *P* = .044) (Table 2). No case of recurrence was observed in patients who achieved complete fistula healing in either group during the follow-up period.

**Table 2:** Postoperative outcomes in the patients, with or without PDRN administration.

	PDRN (n = 21)	Non-PDRN (n = 26)	*P* value
**Rate of complete fistula closure**	16 (83.3)	13 (50.3)	.005^a^
** 6 mo**	14 (70.0)	7 (26.9)	
** 12 mo**	16 (83.3)	12 (46.2)	
**Closure time, mo**	3.3 ± 2.6	5.9 ± 3.8	.044
**Follow up interval, mo**	19.2 ± 11.8	39.4 ± 22.9	.001

Values are n (%) or mean ± SD.

Abbreviation: PDRN, polydeoxyribonucleotide.

^a^The *P* value for the rate of complete fistula closure and fistula recurrence was determined using the log-rank test.

### Factors associated with complete fistula closure

As shown in Table 3, PDRN administration was significantly associated with an increased rate of complete fistula closure in the univariate analysis (HR, 2.647; 95% CI, 1.253-5.591; *P* = .011). In the multivariate analysis, which included 5 confounding factors—age, sex, fistula type, CRP, and PDRN administration—PDRN administration (HR, 2.336; 95% CI, 1.092-4.997; *P* = .029) was identified as the only significant factor associated with faster complete fistula closure. The simple fistula type and lower CRP levels demonstrated a trend toward faster complete fistula closure without statistical significance.

**Table 3:** Factors associated with complete fistula closure.

	Univariate HR (95% CI)	*P* value	Multivariate HR (95% CI)	*P* value
**Age**	1.012 (0.975-1.050)	.525	1.024 (0.982-1.068)	.265
**Sex**	1.193 (0.510-2.795)	.684	0.670 (0.262-1.718)	.405
**Fistula type**		.408		.084
** Simple**	Reference		Reference	
** Complex**	0.734 (0.353-1.527)		0.505 (0.233-1.096)	
**CRP**	0.454 (0.200-1.031)	0.059	0.402 (0.158-1.026)	.057
**PDRN administration**	2.647 (1.253-5.591)	0.011	2.336 (1.092-4.997)	.029

Abbreviations: CI, confidence interval; CRP, c-reactive protein; HR, hazard ratio; PDRN, polydeoxyribonucleotide.

### Subgroup analysis

In the subgroup analysis, the PDRN group demonstrated a significantly higher fistula closure rate in patients who underwent anal fistulotomy or anal fistulectomy than the non-PDRN group (85.7% vs 33.3% at 6 months, 100% vs 50% at 12 months; *P* = .023) (Table 4). Similarly, in cases involving IOC, the PDRN group exhibited favorable outcomes, although the difference did not reach statistical significance (61.5% vs 25.0% at 6 months, 69.2% vs 45.0% at 12 months; *P* = .093).

**Table 4:** Subgroup analysis of fistula closure rates stratified by operation name, with or without administration of PDRN.

Fistulotomy and fistulectomy	PDRN (n = 7)	Non-PDRN (n = 6)	*P* value
**Rate of complete fistula closure**	7 (100.0)	3 (50.0)	.023
** 6 mo**	6 (85.7)	2 (33.3)	
** 12 mo**	7 (100.0)	3 (50.0)	

IOC	PDRN (n = 14)	Non-PDRN (n = 20)	*P* value
**Rate of complete fistula closure**	10 (69.2)	10 (50.0)	.093
** 6 mo**	8 (61.5)	5 (25.0)	
** 12 mo**	10 (69.2)	9 (45.0)	

Abbreviations: IOC, internal opening closure; PDRN, polydeoxyribonucleotide.

## Discussion

Improving the surgical outcomes in patients with CPF remains a significant challenge for colorectal surgeons. Despite advancements in combination therapy with anti-TNF agents, fistula healing rates in CD have only reached approximately 50%.^25^ Surgical intervention continues to play a pivotal role in the multidisciplinary approach to CPF management, underscoring the need for adjunctive strategies that enhance postoperative outcomes. At our institution, we focused on PDRN, which has been recognized for its dual roles as an anti-inflammatory and tissue repair agent, both of which are essential for fistula healing. In this study, the administration of PDRN around the fistula tract correlated with a significantly higher postoperative healing rate of CPF than in cases in which PDRN was not administered. Furthermore, the PDRN injections significantly reduced the follow-up interval required for postoperative monitoring, with a shorter time to achieve complete fistula closure. Adjunctive regenerative strategies such as PDRN may have potential clinical relevance within the current treatment algorithm. Notably, this study represents the first investigation of the feasibility of PDRN in CPF surgery, emphasizing its potential as a promising therapeutic option for patients with CPF.

PDRN is widely used in various clinical fields, offering a practical approach for tissue repair and wound healing. The safety profile of PDRN has contributed to its growing popularity, primarily owing to its selective action as an adenosine receptor agonist that does not exert adverse effects on the immune system.^26^ PDRN facilitates wound healing by enhancing fibroblast and preadipocyte activity through activation of adenosine A2A receptors.^27^ It also promotes the secretion of growth factors, including vascular endothelial growth factor, as well as anti-inflammatory cytokines.^13^ In the context of CPF, the anti-inflammatory and cell-proliferative properties of PDRN may offer substantial therapeutic benefits during fistula surgery. Moreover, PDRN has demonstrated favorable clinical outcomes through relatively safe and simple procedures, such as local injection or topical application in cases of periodontitis.^28^ Given the recurrent and persistent nature of CPF, the simplicity of PDRN administration and its low incidence of side effects, even with frequent use, make it particularly suitable for this condition.

The closure rate of CPF is influenced by the presence of rectal involvement and the complexity of the perianal fistula.^5^^,^^29^ Patients without rectal involvement exhibit higher closure rates than those with rectal involvement. Additionally, complex perianal fistulas are associated with lower closure rates. In our study population, the use of PDRN injection was the only significant factor associated with higher fistula closure rates. The lack of correlation between fistula closure rates and the presence of proctitis or the complexity of the fistula type may be attributed to the small sample size in our study. However, we showed that lower CRP levels were associated with higher fistula closure rates despite the lack of statistical significance. Elevated CRP levels are commonly associated with advanced inflammation and extensive colonic and rectal involvement in CPF cases. While CRP reflects systemic inflammatory burden, any potential benefit of PDRN may be mediated predominantly through modulation of the local wound microenvironment, which may not be captured by systemic biomarkers.

Considering the anti-inflammatory effects of PDRN, CPF cases with varying CD severity may benefit from PDRN injections. In this study, complex fistulas accounted for approximately 60% of the study population in both the PDRN and non-PDRN groups. Our findings suggest that PDRN injection is a feasible treatment option for complicated CPF, yielding favorable outcomes. In the subgroup analysis, a significant correlation was observed between PDRN injection and fistula closure rates in the fistulotomy and fistulectomy groups. Typically, simple fistulas treated with fistulotomy or fistulectomy achieve high closure rates without the need for adjunctive therapy. However, in this study, patients in the PDRN group who underwent IOC similarly exhibited a trend toward improved fistula closure rates compared with the non-PDRN group. These findings suggest that PDRN plays a valuable role in promoting the closure of complex fistulas managed with IOC. Further studies with larger sample sizes are required to achieve a robust statistical significance.

In terms of practicality, PDRN can be administered to patients with CPF in diverse clinical settings, including outpatient clinics and operating rooms, because of its cost-effectiveness and ease of application. This flexibility enables supplementary PDRN injections during postoperative follow-up visits, with or without anesthesia. For optimal administration, we prioritized administering PDRN around the fistula tract prior to suturing the internal opening, as this approach facilitates a more uniform distribution around the target site. The quantity of PDRN administered was determined based on the fistula tract length, guided by the clinical experience with stem cell therapy at our institution.^23^^,^^24^ However, as this quantification was subjectively determined by a single surgeon, further clinical studies, including phase 1 through phase 3 trials, are needed to establish standardized dosing protocols for achieving more favorable outcomes.

The feasibility of PDRN is comparable to that of well-established CPF treatments. To date, the injection of allogeneic or autologous ASCs into the fistula tract is regarded as the most effective and safest strategy for promoting complete healing in CPF surgery. Multiple randomized trials have demonstrated long-term healing rates of approximately 70%, supporting the efficacy of this approach.^30–32^ In our study, PDRN exhibited a complete fistula healing rate comparable to that of stem cells and a reduction in the time required for fistula closure. Although the two agents exhibit comparable efficacy, the cost of stem cell therapy is approximately US$10 500, whereas PDRN is priced at approximately US$90 per 4 ampoules in Korea, making it nearly 100 times more cost-effective. Moreover, PDRN can be administered through a simple and flexible procedure, either during CPF surgery or in an outpatient clinic, via submucosal or intramuscular injection. Its widespread commercial availability further enhances its accessibility and reduces unnecessary patient discomfort. Given its established cost-effectiveness, practicality, and safety profile, PDRN is expected to emerge as a promising therapeutic option for CPF treatment. Following the accumulation of a sufficient number of cases, we plan to conduct further investigations to compare the outcomes of stem cell therapy and PDRN treatment in patients with CPF.

This study had a few limitations. First, its small sample size, short follow-up period, and retrospective design may have introduced a patient selection bias. Second, despite excluding cases involving seton placement, bias due to the heterogeneity of cases involving the 3 different surgical procedures remained unavoidable. Third, the actual healing rate following treatment was not assessed using imaging studies, such as magnetic resonance imaging. Fourth, PDRN administration was determined by a single surgeon at a single center, which may limit generalizability and introduce selection/management bias. Last, follow-up duration differed between groups, with a longer follow-up in the non-PDRN cohort; although we used time-to-event analyses with censoring to mitigate the impact of unequal follow-up, this imbalance may still influence the ascertainment of delayed outcomes. However, patients were selected for each type of surgery according to the universal classification of fistula types, thereby minimizing biases rooted in such limitations.

Despite these limitations, this study suggests the feasibility of using PDRN as a potential therapeutic agent that may facilitate more frequent and accelerated fistula closure following CPF surgery. To our knowledge, this study is the first to explore the potential of PDRN as a treatment option for CPF, highlighting its safety, practicality, and cost-effectiveness. As this retrospective research was designed as a pilot study preceding prospective and randomized trials, further studies with larger sample sizes, extended follow-up periods, and more stringent inclusion criteria are required to validate these findings.

## Data Availability

The datasets analyzed during the present study are available from the corresponding author upon reasonable request.
